# Recurrence or reactivation of SARS-CoV-2 infection after immunosuppressive therapy in patients with ANCA-associated vasculitis and COVID-19 

**DOI:** 10.5414/CNCS110567

**Published:** 2022-01-05

**Authors:** Mansour Mbengue, Bede Bigirimana, Lolly Romeo Irankunda, Mohamed Cherif Dial, Abdou Niang

**Affiliations:** 1 Department of Nephrology, Dalal Jamm University Hospital, and; 2 Department of Anatomy pathological, Idrissa Pouye General Hospital, Dakar, Senegal

**Keywords:** COVID-19, ANCA, vasculitis, recurrence

## Abstract

Abstract. Acute tubular injury is the lesion most frequently described in this disease. However, four cases of ANCA-associated vasculitis (AAV) with COVID-19 with pauci-immune glomerulonephritis have recently been described. We report the case of an African woman, aged 70, in whom we diagnosed an AAV with pauci-immune glomerulonephritis in the context of COVID-19. She was treated with hydroxychloroquine and azithromycin for COVID-19. Corticosteroids and cyclophosphamide have been used for the treatment of vasculitis. The evolution was marked by the reappearance of COVID-19 one month after the beginning of an immunosuppressive therapy. The patient died a week later from respiratory failure. The occurrence of AAV during COVID-19 may not be due an unfortunate association but triggered by infection with SARS-CoV-2. The use of immunosuppressive therapy should be discussed due to the potential risk of reactivation or recurrence of the viral infection.

## Introduction 

Coronavirus disease 2019 (COVID-19) is a new disease that appeared at the end of 2019. It is caused by severe acute respiratory syndrome coronavirus 2 (SARS-CoV-2). COVID-19 is mainly characterized by respiratory manifestations but can affect other organs, in particular the kidneys. However, not every kidney damage due to this disease is fully understood. Acute tubular injury (ATI) and collapsing glomerulopathy are the lesions most frequently described in this disease [1]. However, four cases of antineutrophilic autoantibody (ANCA)-associated vasculitis (AAV) in COVID-19 with pauci-immune glomerulonephritis have recently been described [2]. The occurrence of this form of kidney damage has several implications. First in terms of etiopathogenesis due to the fact that a triggering factor has always been suspected in AAV [3]. There are also therapeutic implications due to the paradox of the use of immunosuppressive therapy in the context of acute viral infection with a risk of reactivation of the virus. Finally, the severity of kidney damage during AAV often leads to resistance to immunosuppressive therapy. We report the case of a patient who presented with AAV and COVID-19 and who contracted COVID-19 again after receiving immunosuppressive therapy. 

## Case presentation 

**Figure 1:**
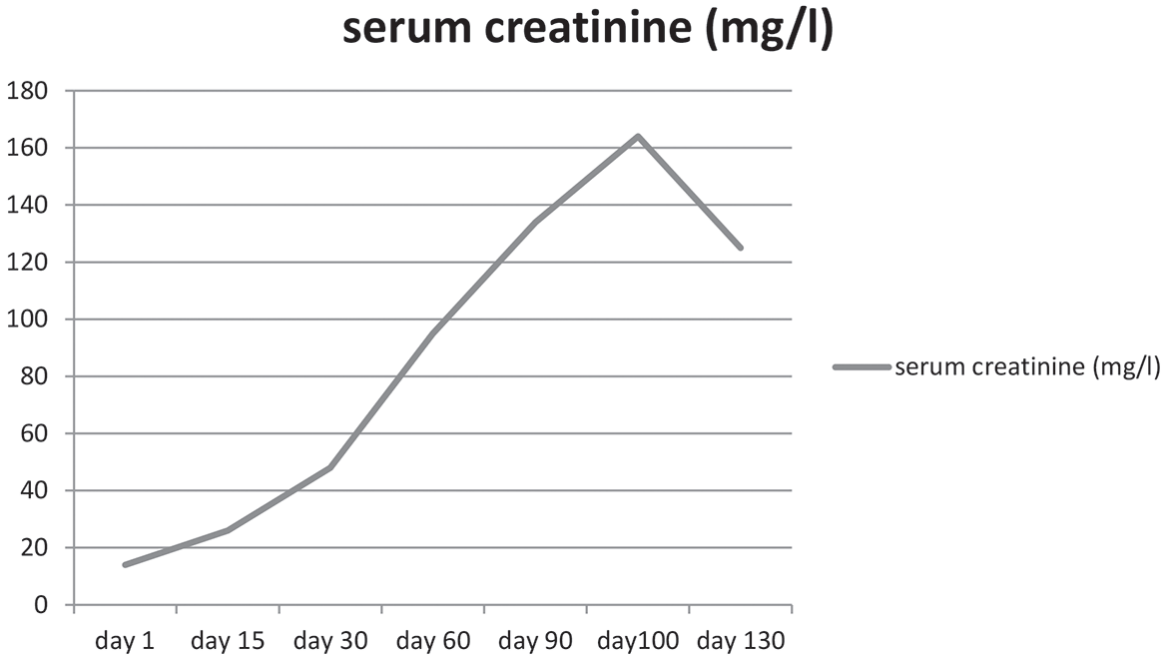
The kinetics of serum creatinine.

**Figure 2:**
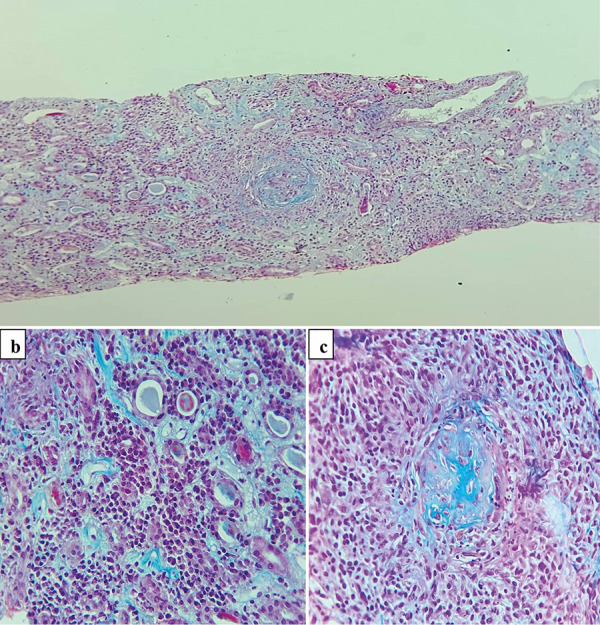
a: Destroyed glomerulus: Residual sequelae in the form of fibrosis of the glomerulus surrounded by a granulomatous polymorphic infiltrate. b: Severe tubulointerstitial lesions with an infiltrate mainly formed of plasma cells, associated with edema and lesions of tubulitis. c: A glomerulus with global fibrosis associated with a peripheral granulomatous epithelioid infiltrate.

We describe a 70-year-old African woman with history of hysterectomy for uterine myomatosis 10 years ago. She was seen in the emergency room with complaints of fever, cough, and rhinorrhea. On physical examination, blood pressure was 120/70 mmHg, heart rate 112 beats/min, temperature 40 °C, respiratory rate 24 cycles/min, and weight 82 kg. The blood count showed a hemoglobin level of 11.2 g/dL, hyperleukocytosis at 19,000/mm^3^, the C-reactive protein was elevated to 260 mg/L. Serum creatinine was normal on admission at 12 mg/L, and blood urea was 0.36 g/L. The diagnosis of COVID-19 was retained by a positive SARS-CoV-2 reverse transcription polymerase chain reaction (RT-PCR). The chest CT scan performed without injection of contrast medium showed ground-glass opacities in both pulmonary fields. The patient was put on hydroxychloroquine (HCQ) 600 mg/day, azythromycin (AZT) 500 mg/day, and dexamethasone 6 mg/day. During hospitalization, the patient presented an acute kidney injury (AKI) with serum creatinine at 24 mg/L then at 36 mg/L. Proteinuria was 0.6 g/day, and urine sediment was active with hematuria at 20 red blood cells per high-power field (RBC/hpf) with hematuria with blood cylinders. Two weeks after diagnosis, the patient was cured of COVID-19 with two negative control PCR tests. The follow-up chest CT scan was normal. However, renal failure persisted with worsening renal impairment. The kinetics of serum creatinine are shown in Figure 1. The worsening of the renal insufficiency prompted us to measure the ANCA which were strongly positive at 80 IU (lab threshold 20) and were of the perinuclear-ANCA (p-ANCA) type with an anti-myeloperoxidase (MPO) specificity. Antinuclear antibody and anti-glomerular basement membrane (anti-GBM) antibody were negative. Kidney biopsy was performed and showed an aspect of crescentic glomerulonephritis with residual sequelae in the form of fibrosis of the glomerulus surrounded by a granulomatous polymorphic infiltrate with severe tubulointerstitial lesions (Figure 2). The diagnosis of AAV with pauci-immune glomerulonephritis was retained. The patient was put on methylprednisolone 10 mg/kg for 3 days then on prednisone 60 mg/day. Cyclophosphamide (CYC) was used as an immunosuppressant at a rate of 500 mg every 3 weeks. After 1 month of treatment, the patient was readmitted to the hospital for a deterioration of general conditions. 48 hours later, the patient presented respiratory distress, and the chest CT scan performed showed ground-glass opacities suggestive of a SARS-CoV-2 infection. The SARS-CoV-2 RT-PCR was positive. This new infection with SARS-CoV-2 had occurred 6 months after the first. The patient was again put on HCQ 600 mg/day, AZT, dexamethasone, and heparin. The patient died a week later from respiratory distress. 

## Discussion 

Currently, it is clearly established that some forms of vasculitis may be secondary to viral infections. This is the case with hepatitis B virus-related polyarteritis nodosa and hepatitis C virus-related cryoglobulinemic vasculitis. For SARS-CoV-2, although the association with vasculitis has not yet been confirmed, the onset of cases of Kawasaki disease related to SARS-CoV-2 infection is an example of SARS-CoV-2 triggering vasculitis [7]. If we look back at the etiopathogenesis of AAV, the existence of an environmental or infectious triggering factor has been implicated to explain the origin of ANCA. The preexistence of natural ANCA which become pathogenic following various events such as exposure to exogenous antigens, ectopic or abnormal expression of the target autoantigens of ANCA, or a dysfunction of the regulatory cells controlling the tolerance of ANCA antigens [3]. ANCA are directed against MPO and PR3 which are enzymes present in polynuclear neutrophils [8]. More recently, it has been demonstrated that neutrophil extracellular traps (NETs) serve as a source of autoantigens presenting MPO and PR3 to the immune system. COVID-19 and AAV are associated with the formation of NETs, which may explain the development of autoimmunity in the context of this acute viral infection [6]. 

In terms of clinical and biological manifestations, there is no difference between our patient and four other cases already described. We observed that the association of COVID-19 and AAV can occur regardless of age or gender. Our patient did not have extrarenal clinical manifestations of vasculitis. When we reviewed the cases already described in the literature, only one presented an extrarenal sign such as arthritis [7]. Intra-alveolar hemorrhage was found in two cases in the literature but could not be directly related to vasculitis because it can also be caused by COVID-19 [7, 8]. Our patient had ANCA with a specific anti-MPO, but according to the literature ANCA with a specific anti-PR3 can be observed in this association (Table 1). 

The treatment remains the conventional treatment of AAV, based on corticosteroids associated with an immunosuppressant, either CYC or rituximab (RTX) [2]. This differentiates it from other types of post-infectious vasculitis which do not require an immunosuppressive treatment, and which often heal with treatment for the infection associated, with low corticosteroid therapy. In our patient, renal failure continued to worsen despite recovery from COVID-19, which suggests that SARS-CoV-2 is not a direct cause of AAV but a triggering factor. For the four cases of AAV in COVID-19 that were published, RTX or CYC or intravenous immunoglobulin (IVIG) were used. RTX was used in two patients and CYC in one patient and the outcome was favorable with renal remission [2]. 

The reappearance of COVID-19 in our patient raises two questions. First, we can think of a mutation of the virus, a reinfection by another strain of the SARS-CoV-2. Second, we can ask whether the virus persisted in the body after recovery from the disease. That is, the virus remained in the body in a latent state as in some viral infections such as Epstein-Barr virus infection and infection with *Herpesviridae*. Immunosuppressive treatment could lead to reactivation of SARS-CoV-2, which would have remained in the latent phase. However, it should be noted that in the four published cases of AAV and COVID-19, a reappearance of COVID-19 was not noted in any patient. 

## Conclusion 

The occurrence of AAV during COVID-19 may not be a random association but triggered by infection with SARS-CoV-2. The use of immunosuppressive therapy should be discussed because of the potential risk of reactivation of the viral infection. 

## Statement of ethics 

The present case report adhered to the Declaration of Helsinki. Written informed consent for publication was obtained from the patient’s son. 

## Funding 

This research received no specific grant from any funding agency in the public, commercial, or not-for-profit sectors. 

## Conflict of interest 

The authors have no conflict of interest to declare. 

**Table 1.** Demographic, clinical, therapeutic and outcome characteristics of published case reports.

Case report	Age	Gender	Ethnicity	Vasculitis renal manifestations	Vasculitis extrarenal manifestations	ANCA type	Kidney pathology	Covid-19 treatment	RRT	Vasculitis treatment	Outcome
Moeinzadeh et al. [8]	25	M	Unspecified	AKI, proteinuria, hematuria	Alveolar hemorrhage	PR3	Crescentic GN	HCQ, levofloxacin	No	Corticosteroids + CYC + PLEX + IVIG	AKI and COVID-19 in recovery
Uppal et al. [1]	64	M	African American	AKI, proteinuria, hematuria	None	PR3	Crescentic GN	Convalescent plasma, tocilizumab	IHD	Corticosteroids + RTX	AKI and COVID-19 in recovery
Uppal et al. [1]	46	M	Asian	AKI, proteinuria	Leukocytoclastic vasculitis	MPO	Focal necrotizing GN	HCQ, Azithromycin	No	Corticosteroids + RTX	AKI and COVID-19 in recovery
Hussein et al. [7]	37	F	Middle Eastern	AKI, proteinuria	Arthritis alveolar hemorrhage	PR3		Ritonavir/lopinavir	US	Corticosteroids + PLEX + IVIG	Patient died
Our patient	70	F	African	AKI, proteinuria, hematuria	None	MPO	Focal necrotizing GN	HCQ, azithromycin	IHD	Corticosteroids + CYC	Patient died

AKI = acute kidney injury; ANCA = antineutrophilic autoantibody; MPO = myeloperoxidase; PR3 = proteinase 3; GN = glomerulonephritis; HCQ = hydroxychloroquine; RRT = renal replacement therapy; IHD = intermittent hemodialysis; CYC = cyclophosphamide; PLEX = plasmapheresis; IVIG = intravenous immunoglobulin; US = unspecified.
